# Hyperammonemic Encephalopathy: A Unique Presentation of Multiple Myeloma

**DOI:** 10.7759/cureus.12781

**Published:** 2021-01-19

**Authors:** Steven Douedi, Shruti Kapadia, Mohammed AlAzzawi, Shuvendu Sen

**Affiliations:** 1 Internal Medicine, Jersey Shore University Medical Center, Neptune, USA; 2 Internal Medicine, Jersey Shore University Medical Center, Neptune, USA

**Keywords:** multiple myeloma, cancer, hyperammonemia, encephalopathy, plasma cell, malignancy

## Abstract

Multiple myeloma is a malignancy of plasma cells which are commonly found in the bone marrow. Known for causing a wide range of symptoms and affecting various organ systems, multiple myeloma is a rare malignancy with the entire pathophysiological process yet to be elucidated. We present a case of a 51-year-old male with a history of previously treated multiple myeloma in remission, initially presenting with chest pain with an unremarkable work-up. His hospital course was complicated by hyperammonemia causing encephalopathy requiring mechanical ventilation. After an extensive work-up to find an underlying cause, he was diagnosed with relapsing multiple myeloma. Due to a prolonged and complicated hospital course, the family pursued comfort measures and the patient passed away peacefully. Multiple myeloma induced hyperammonemic encephalopathy is a rare phenomenon carrying a high morbidity and mortality rate. Being still poorly understood, this manifestation of an already lethal diagnosis should be considered as a differential diagnosis of hyperammonemia. While early and aggressive treatment has shown some benefit and improved patient outcomes, further studies and understanding is needed to help diminish the mortality associated with hyperammonemic encephalopathy due to multiple myeloma.

## Introduction

Multiple myeloma is a plasma cell disorder found to constitute up to 10% of all hematologic cancers and has been found to primarily affect African Americans and males [1,2]. Suggested to be linked to a pre-malignant stage known as monoclonal gammopathy of undetermined significance (MGUS), multiple myeloma pathophysiology is still poorly understood [2]. Symptoms revolve around the affected organ systems and commonly include fatigue, bone pain, abdominal pain, urine loss, and weight loss [2]. We herein present a case of a middle-aged Caucasian male presenting with chest pain ultimately found to have hyperammonemia leading to encephalopathy in the setting of recurrent multiple myeloma.

## Case presentation

A 51-year-old Caucasian male with a medical history of coronary artery disease and multiple myeloma diagnosed six years ago presented to the emergency department (ED) complaining of nonradiating generalized chest pain that has been progressively worsening for one week. His multiple myeloma was in remission after he received treatment with four cycles of chemotherapy with ninlaro, darzalex, and venetoclax and stem cell transplant two years ago. He has since been started on daratumumab weekly with chronic dexamethasone. In the ED, his physical examination was unremarkable. Electrocardiogram (EKG) was unremarkable without ST-T segment changes and cardiac troponins were within normal limits (normal value: <0.04 ng/dL). Laboratory results revealed a hemoglobin level of 8.0 g/dL, white blood cell count of 3.2 10*3/uL, and platelet count of 20 10*3/uL. His aspartate aminotransferase (AST) level was 30 U/L and alanine aminotransferase (ALT) was 26 U/L. Vitals in the ED were a blood pressure of 117/70 mm Hg, heart rate of 120 beats per minute, temperature of 97.8 degrees Fahrenheit, respiratory rate of 18 breaths per minute and oxygen saturation of 100% on room air. He urgently received two units of platelets while in the ED. Hematology and oncology was consulted for possible disease reoccurrence. 

**Figure 1:**
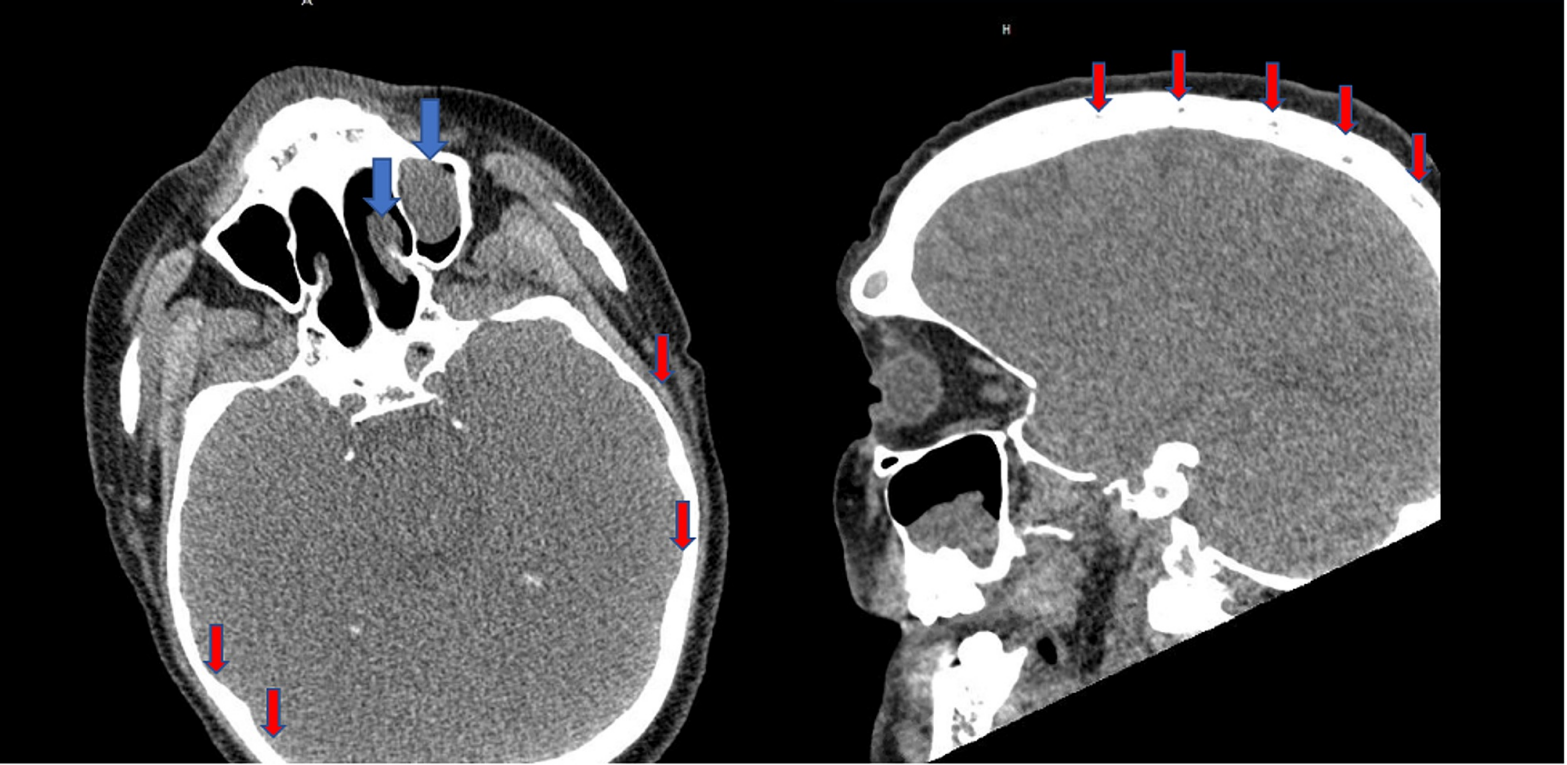
CT scan of head and sinuses. Complete opacification of the left frontal sinus and left frontal, maxillary and sphenoid sinus disease with small mucous retention cysts in the right sphenoid sinus and leftward nasal septal deviation is seen (blue arrows) and innumerable mixed lytic and sclerotic lesions within the calvarium suspicious for multiple myeloma (red arrows).

While on the medical floors his platelet and hemoglobin levels continued to decrease, and he received several additional transfusions to maintain hemoglobin above 8.0 g/dL and platelet count >20 10*/uL. Six days after admission the patient was noted to become more confused and his mental status began to decline, a computed tomography (CT) scan of the head and CT of the sinuses showed complete opacification of the left frontal sinus and left frontal, maxillary and sphenoid sinus disease with small mucous retention cysts in the right sphenoid sinus, leftward nasal septal deviation seen, and innumerable mixed lytic and sclerotic lesions within the calvarium suspicious for multiple myeloma (Figure 1). Protein panel for suspicious multiple myeloma was also obtained (Table 1). An ammonia level at that time was 118 umol/L (normal levels: 9-35 umol/L). The infectious disease team started the patient on 1 gram of meropenem every eight hours and acyclovir 10 mg/kg every eight hours for possible meningitis however a lumbar puncture was unable to be safely performed due to a low platelet count. Two days after admission, he became more obtunded, tachypneic (30-40 breaths per minute), tachycardic (heart rate 140-150 beats per minute) and was eventually intubated and transferred to the intensive care unit (ICU) for further management. 

**Table 1:** Protein levels showing increased alpha globulins and kappa light chains suspicious for multiple myeloma.

Laboratory test	Value	Reference range	Units
Total Protein	5.4	6.0-8.0	g/dL
Alpha 1 Globulins	0.71	0.1-0.4	g/dL
Alpha 2 Globulins	1.31	0.6-1.0	g/dL
Beta Globulins	0.84	0.60-1.30	g/dL
Gamma Globulins	0.29	0.70-1.50	g/dL
Kappa Qt Free Light Chains	130.00	3.30-19.40	mg/L
Lambda Qt Free Light Chains	<2.40	5.71-26.30	mg/L

A lumbar puncture was ultimately obtained with a dry tap. Ammonia level at this time was 256 umol/L and AST was 41 U/L and ALT was 45 U/L. CT scan of the abdomen and pelvis was unremarkable and hepatitis panel was negative. The patient was started on lactulose and rifaximin for hyperammonemia. A bone marrow biopsy was performed and resulted in showing markedly hypercellular marrow about 100% diffusely infiltrated by plasma cells (>70% of cellularity). Sheets of neoplastic plasma cells, frequent bi- or tri-nucleated forms, some plasma cells with prominent nucleoli, and increased mitotic activity were also noted on the biopsy. The overall morphologic features of plasma cells were noted to be intermediate to high histologic grade and maturing trilineage hematopoiesis were markedly reduced. Immunostains were performed on the biopsy with the following results: CD138: Highlights numerous plasma cells; involving more than 70% of bone marrow cellularity. P53: Strong nuclear expression of p53 in >60% of plasma cells (overexpressed). Ki67: High proliferation rate in plasma cells (70% nuclear expression). He was ultimately diagnosed with a relapse of his multiple myeloma. Despite being aggressively managed in the ICU, he remained on the ventilator and was unable to be weaned off by day 14. Due to a poor prognosis, the family ultimately decided to pursue palliative and comfort care.

## Discussion

Multiple myeloma is an aggressive plasma cell cancer with increasing incidence diagnosed on bone marrow biopsy demonstrating >10% plasma cells [1,2]. In addition to biopsy findings, multiple myeloma must have the presence of serum or urinary monoclonal proteins and evidence of end-organ damage such as anemia, hypercalcemia, or renal insufficiency [2]. Imaging studies such as X-ray skeletal surveys, CT scans, and MRI can be used to detect and increase suspicion of multiple myeloma with typical features of lytic lesions which can be detected in up to 80% of individuals [3,4]. Treatment for multiple myeloma revolves around combination chemotherapy and ultimately stem cell transplantation [2,4]. 

While multiple myeloma can present with a wide range of symptoms and may be asymptomatic in some cases, patient’s commonly experience fatigue, weight loss, and bone pain [2]. Fatigue due to anemia occurs in up to 70% of patients with multiple myeloma; renal dysfunction (50%) and hypercalcemia (25%) are also commonly found on laboratory testing [2]. Uncommonly, high levels of ammonia leading to encephalopathy have been reported in multiple myeloma [5,6,7]. While the pathophysiology of hyperammonemia encephalopathy in patients with multiple myeloma is poorly understood, it is suspected multiple myeloma can lead to hepatic failure leading to these increased levels [8,9]. In the absence of liver dysfunction, studies suggest either aggressive myeloma cell lines can produce ammonia or myeloma-related humoral factors influence amino acid metabolism leading to an increase in ammonia levels, as possibly seen in our patient [8,9]. Nonetheless, the finding is associated with a high mortality and worse patient outcomes [6,9,10]. Lora-Tamayo et al. reported 22 out of 25 patients had lower ammonia levels when treated with combined aggressive chemotherapy with 15 of the 25 ultimately surviving the episode and an overall mortality of 44% [9]. Pham et al. reported similar findings, with a 40% inpatient mortality in patients who received chemotherapy and a 75% mortality in those who did not [11]. 

Our patient presented in this case was found to have an ammonia level reaching 256 umol/L and was encephalopathic eventually succumbing to his condition, consistent with previous studies showing poor outcomes. Clinicians are urged to keep late-stage multiple myeloma in mind as a differential diagnosis and cause of hyperammonemia.

## Conclusions

This report illustrates the rare and infrequent hyperammonemic encephalopathy due to underlying multiple myeloma that failed mechanical ventilation and ultimately succumbing to the condition supported by published data depicting poor outcomes in such patients. In summary, clinicians should be aware of and keep multiple myeloma in the differentials with hyperammonemia. While chemotherapy has shown some response and benefit in decreasing ammonia levels due to multiple myeloma, the prognosis remains poor.
